# Extramedullary Tibial Guide Alignment Is Not Affected by Excess Lower Limb Fat Distribution in Total Knee Arthroplasty

**DOI:** 10.7759/cureus.24443

**Published:** 2022-04-24

**Authors:** John T Williams, Rajat Varma

**Affiliations:** 1 Trauma & Orthopaedics, King’s College Hospital, London, GBR

**Keywords:** arthroplasty, knee, anthropometrics, obesity, osteoarthritis

## Abstract

Background

The goal of this study is to investigate whether excess lower limb fat distribution affects tibial guide alignment in conventional total knee arthroplasty (TKA) with extramedullary guides. A thicker soft tissue envelope may affect the accuracy of extramedullary cutting guide placement and subsequent instrumentation. Previous studies have used body mass index (BMI) to stratify patients, a poor proxy of lower limb fat distribution, which may explain conflicting results reported on this topic to date. This study overcomes this issue by using a novel, radiographic anthropometric index to assess lower limb fat distribution.

Methodology

This is a single-surgeon, single-implant, single-centre retrospective series of 102 consecutive primary TKAs. The suprapatellar fat index (SPFI) and BMI were recorded for all patients, and postoperative tibial component alignment measurements were calculated. Secondary outcome measures included femoral component alignment, femorotibial alignment, length of hospital stay, tourniquet time, blood loss, and complications/reoperations.

Results

In this study, 102 patients (average age of 69) had an average BMI of 30.8 kg/m^2^ (19.2-45.5 kg/m^2^) and an average SPFI of 0.26 (0.09-0.57). Multiple regression analysis demonstrated that increasing leg fat distribution did not affect tibial component alignment in the coronal or sagittal plane.

Conclusions

Excess lower limb fat distribution, simply measured using the SPFI*, *does nothave a significant effect on tibial component positioning when extramedullary guides are used in conventional TKA.

## Introduction

Component malalignment [1,2] and obesity [3-6] are independent predictors of poor outcomes in total knee arthroplasty (TKA). When in combination, the risk of early revision is greatly increased [1]. There is conflicting evidence regarding whether obesity itself is a risk factor for component malalignment. The proposed mechanism is that excess adipose tissue in obese patients increases the technical demands of accurate exposure, instrumentation, and cutting guide placement [7,8]. A thicker soft tissue envelope may cause obscuration of bony landmarks, which, in conventional TKA, is of particular relevance when extramedullary alignment guides are utilised for the tibia. This issue is of less relevance to femoral alignment guides which are generally intramedullary and utilise exposed bony landmarks and pre-determined cut angles.

Previous studies have used body mass index (BMI) to define obesity when investigating the risk of component malalignment [8-14]. Obesity is defined as a BMI of >30 kg/m^2^. However, BMI is not a measure of body fat distribution [15] and as such has limited predictive value in assessing the technical challenge of TKA. For example, patients with central or truncal obesity may have a BMI of >30 kg/m^2^ but relatively little fat distribution in the lower limbs.

To overcome this issue, this study stratifies patients undergoing TKA according to lower limb fat distribution, calculated by a simple, novel, radiographic anthropometric index. Our study hypothesises that excess lower limb fat distribution is associated with tibial component malalignment. We are unaware of any other study that has used an anthropometric measurement to directly investigate the issue of postoperative component or limb malalignment in TKA. Secondary outcome measures include femoral component alignment, femorotibial alignment, tourniquet time, blood loss, length of hospital stay, and complications.

## Materials and methods

Local registry data for consecutive primary TKAs performed by a single senior knee surgeon between January 2017 and December 2019 were reviewed retrospectively. All surgeries were performed in an elective orthopaedic centre affiliated with a large tertiary hospital. Exclusion criteria were complex primary TKA, previously retained hardware, or inadequate pre or postoperative imaging. Malrotated anteroposterior (AP) radiographs in which the patella was not centrally positioned over the femur were excluded to ensure consistent width measurements between patients.

In total, 136 patients were identified in a local registry search, of whom 102 were suitable for inclusion in the study. Patient demographics, body measurements, procedure details, length of hospital stay, tourniquet time, blood results, complications and reoperation data were collected from electronic records. BMI of the patients was recorded at the preoperative assessment appointment within four weeks of surgery.

**Figure 1:**
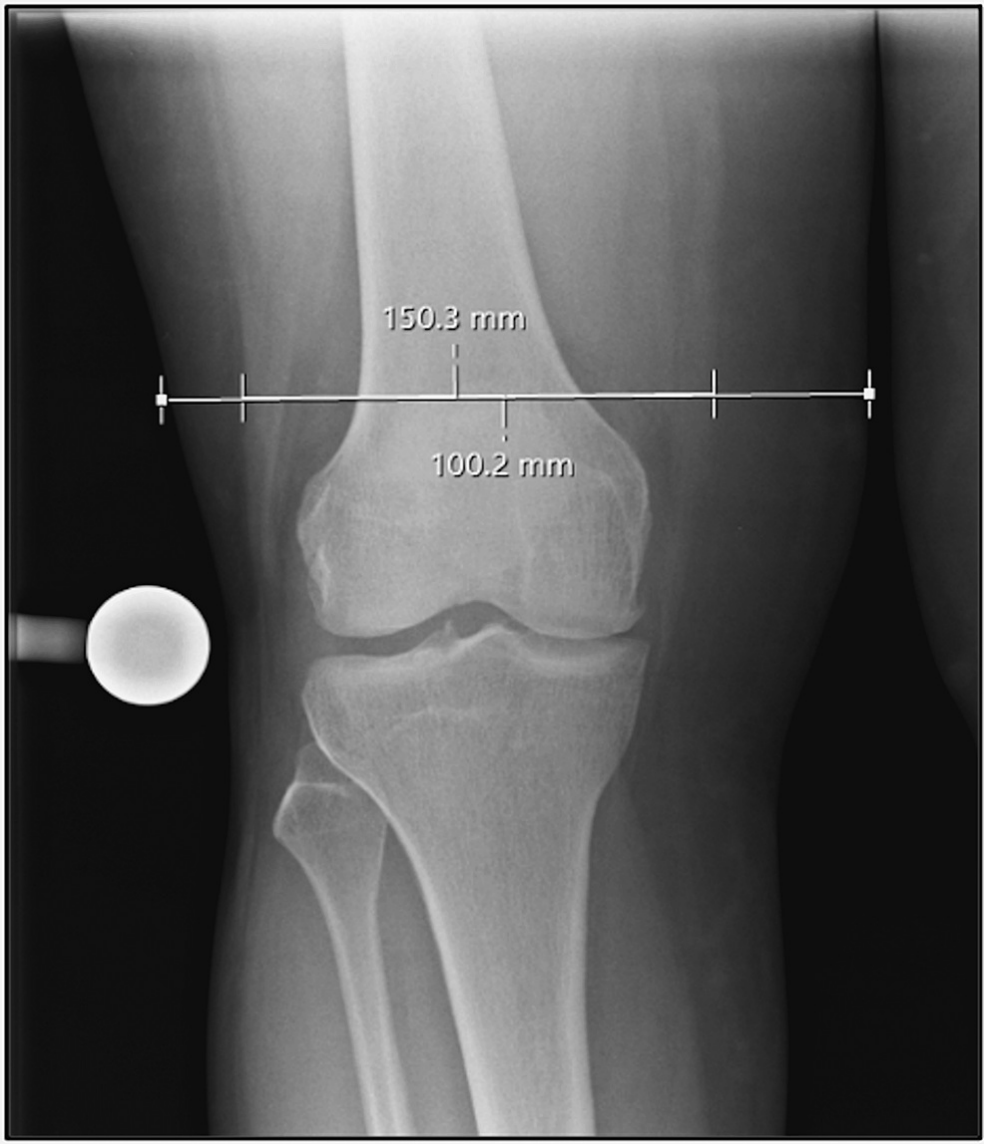
Suprapatellar fat index measurement on weight-bearing anteroposterior knee radiograph.

Preoperative radiographs were reviewed for each patient, and radiographic anthropometric measurements were recorded (Figure 1). First, a horizontal measurement of limb width at the level of the superior pole of the patella on an AP knee radiograph was made. A second horizontal measurement at the same level was made from the lateral fat-muscle interface to the medial fat-muscle interface. The proportion of the horizontal diameter of the limb represented by subcutaneous fat was then calculated, referred to hereafter as the suprapatellar fat index (SPFI).

**Figure 2:**
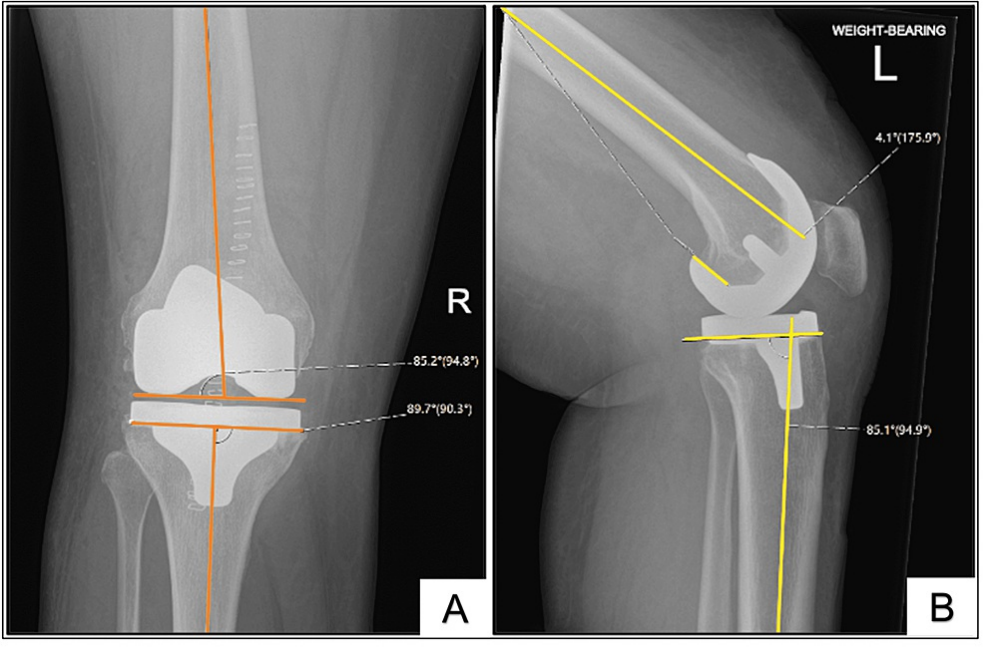
(A) Coronal femoral and tibial component alignment, and (B) sagittal femoral and tibial component alignment.

Postoperative radiographs were assessed for knee and component alignment using the Knee Society Radiographic Evaluation System [16]. The following four component alignment measures were recorded: coronal and sagittal tibial and femoral component alignment (Figure 2). Postoperative femorotibial angle was also recorded. All knee radiograph series were standard weight-bearing AP and supine laterals at 30 degrees of flexion, and measurements were performed on digitised films using Sectra PACS radiology software (Sectra, Linköping, Sweden).

Surgery was performed by a single senior knee surgeon utilising the same surgical technique and Triathlon® implant (Stryker, Kalamazoo, MI, USA). All surgeries were performed under a tourniquet utilising a medial parapatellar approach. Tibial preparation was performed using an extramedullary alignment guide and femoral preparation with an intramedullary guide. Guides were applied to the limb and cuts were referenced, as described in the manufacturer’s surgical protocol [17]. All implants were cemented and of cruciate-retaining design. The surgical aim was to align components perpendicular to their respective mechanical axis and achieve neutral lower limb mechanical alignment.

Means and standard deviations were calculated for SPFI, BMI, and component alignment measurements. Multiple regression analysis was performed to examine the effect of SPFI and BMI on tibial component positioning. This was repeated for the secondary outcome measures of femoral component alignment, femorotibial alignment, blood loss, tourniquet time, and length of stay. All statistical tests were two-sided, and p-values of <0.05 were considered statistically significant. Statistical analysis was performed using GraphPad (San Diego, USA).

## Results

**Figure 3:**
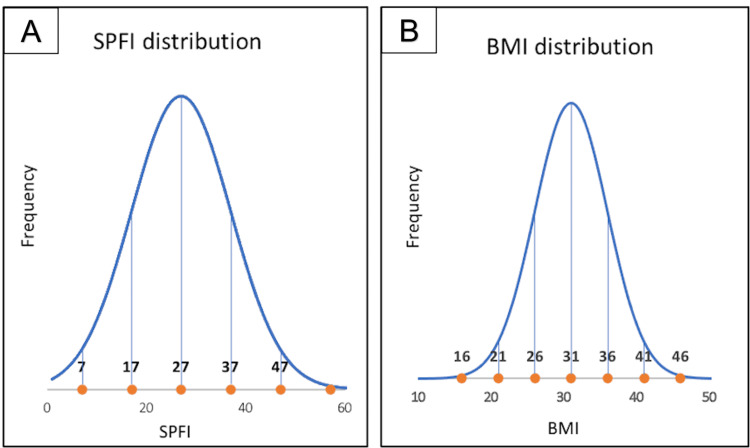
Normal distribution graphs of the patients. A: SPFI; B: BMI. SPFI: suprapatellar fat index; BMI: body mass index

Following exclusions, 102 patients met the eligibility criteria for the study, including 70 female and 32 male patients, with a mean age of 69 (range = 48-86 years) years at the time of the surgery. Mean BMI was 30.8 kg/m^2^ (range = 19.2-45.5 kg/m^2^), with 53 patients classified as obese (BMI = 30 kg/m^2^) and 49 as non-obese (BMI = <30 kg/m^2^). The mean SPFI was 0.26 (0.09-0.57). Figure 3 demonstrates normal overall patient distribution by SPFI and BMI. The average tibial component positioning was 0.7° valgus with a 5.4° posterior slope. The average femorotibial angle was 5.9° valgus (range 0.3-11); the average femoral component positioning was 5.9° valgus to the femoral anatomic axis and 2.1° of flexion.

Multiple regression analysis (Table 1) demonstrated that increasing SPFI did not significantly affect either sagittal (p = 0.41) or coronal tibial component alignment (p = 0.33). BMI did not affect tibial component alignment.

**Table 1:** Multiple regression analysis of SPFI against variables. SPFI: suprapatellar fat index

	r^2^	Coefficient	95% CI lower	95% CI upper	P-value
Coronal tibial component alignment	0.061	-0.020	0.070	0.029	0.415
Coronal femoral component alignment	0.005	0.014	-0.033	0.060	0.554
Sagittal tibial component alignment	0.011	0.020	-0.025	0.066	0.373
Sagittal femoral component alignment	0.063	-0.058	-0.107	-0.008	0.024
Femorotibial angle	0.116	0.087	0.032	0.143	0.002
Blood loss	0.004	-0.016	-0.179	0.148	0.850
Length of stay	0.031	0.062	-0.017	0.141	0.123
Tourniquet time	0.178	-0.061	-0.550	0.428	0.802

Regarding secondary outcome measures, increasing SPFI was associated with valgus femorotibial malalignment (p = 0.002) and femoral component extension (p = 0.015). SPFI and BMI did not have a significant association with any other alignment metrics. Neither BMI nor SPFI significantly affected blood loss, tourniquet time, or length of stay.

There were seven recorded complications in seven separate patients. Five patients underwent manipulation under an anaesthetic for postoperative stiffness, one had a superficial wound infection, one had a prosthetic joint infection, and there was one vessel injury requiring vascular repair.

## Discussion

This study has demonstrated that there is no association between increasing lower limb fat distribution and tibial component malalignment. This finding supports the use of extramedullary tibial guides as a safe and accurate method for component alignment in all sizes of lower limbs.

BMI is the most commonly used method for assessing obesity due to its simplicity, low cost, and near-universal recognition. However, it has two significant and well-recognised limitations. It does not account for body fat distribution and does not differentiate between fat mass and fat-free mass [15]. Therefore, its utility as a proxy measure for the subcutaneous fat thickness of the lower limb is limited.

The radiographic anthropometric index utilised in this study, while not without limitation, circumvents the above two issues, allowing an estimate of fat versus fat-free mass with specific relevancy to the surgical site. In addition, it is easily reproducible and does not require further measurements or investigation beyond a routine preoperative knee radiograph. The main limitation of the index is the assumption that the two-dimensional measurement of fat proportion on an AP radiograph is proportional to the overall subcutaneous fat thickness of the lower leg, the extent of which may vary between individuals. A further measure of radiographic fat thickness at the ankle may have been relevant, although is not routinely included in TKA patients at our institute.

Obesity as a risk factor for limb and component malalignment after TKA has been previously investigated, and conflicting results have been reported [8-14]. The use of BMI to stratify patients, in addition to the high heterogeneity of study design and results reporting, contributes to the disagreement. Furthermore, limb and component malalignment are frequently reported as a secondary outcome measure without including adequate methodology or quantitative data.

Estes et al. reported that increased BMI was associated with a higher rate of postoperative limb malalignment in a retrospective study of 196 conventional TKAs with full-length standing radiographs [7]. Krushell and Fingeroth reported increased rates of varus tibial and femoral component malalignment in a study of 39 patients with a BMI of >40 kg/m^2^ and 39 matched patients with a BMI of <30 kg/m^2^ [9]. Kamat et al. also found an increased rate of tibial component malalignment in 74 obese patients operated with standard instrumentation [14].

Compton et al. recently investigated the relationship between obesity and tibial component alignment and found no differences between the obese group and the non-obese group [8]. Similarly, Amin et al., in a prospective study of limb and component alignment in 41 conventional TKAs performed in the non-obese and morbidly obese patients, found no difference between the groups [10].

Two studies have used anthropometric measurements around the knee to investigate associated outcomes in TKA, although not component or limb alignment. Armstrong et al. reported that knee-ankle circumference and incisional fat thickness were better predictors of tourniquet time, a surrogate of surgical difficulty, in TKA than BMI [18]. Watts et al. measured pre-patellar and pre-tibial fat thickness on lateral radiographs and found an increased association with wound complications and infections in TKA in the morbidly obese [19].

The continuing disagreement on this topic may represent the failure of previous studies to utilise a lower limb anthropometric measurement instead of BMI to investigate limb and component alignment, despite soft tissue interference at the surgical site being the key point of interest. We believe this is the first study to directly investigate the relationship between lower limb fat distribution and tibial component alignment in conventional TKA.

Our results do not demonstrate a correlation between excess fat distribution in the lower limb and tibial component malalignment when using extramedullary guides in a cohort of patients with above-average BMI matched for sex and age [20]. This suggests that extramedullary tibial guides may be used safely and with satisfactory accuracy even in larger limbs.

The unexpected association of increased fat thickness with femoral component extension is unclear. This may be due to increased difficulty in exposing the distal femur and flexing the knee, although the intramedullary nature of the femoral alignment guides makes this less likely. The propensity for larger legs to result in slight postoperative valgus limb malalignment may be due to an additive effect of minor component valgus malpositioning, not adequately powered for in this study, although preoperative alignment also likely plays a role.

Limitations of this study include the lack of hip to ankle standing radiographs as this is not a part of the standard protocol for primary TKA in our institution. Including this in future studies will allow the calculation of true mechanical alignment rather than the use of the femorotibial angle as a proxy. Furthermore, as a single-surgeon series, the results of this study may not be applicable equally to all surgeons.

Whether achieving neutral mechanical alignment is superior to restoring the anatomic or kinematic alignment of the knee is a topic frequently debated in the literature [21], and alternative surgical techniques, such as patient-specific cutting guides, computer-navigated, and robotic-assisted surgery, are gaining popularity. It is widely accepted that these alternative methods permit increased accuracy of component positioning, even in obese patients [14,22,23], although clinical and patient-reported outcome improvements are less clear. It is possible that increased adipose tissue and difficult exposure may also affect the accuracy of the placement of patient-specific guides and navigation referencing. Soft tissue artefacts on preoperative radiographic studies have also been cited as a potential issue in the manufacturing of custom cutting blocks in obese patients [7].

## Conclusions

This study has demonstrated that extramedullary tibial alignment guides may be used reliably in conventional TKA in patients with excess lower limb fat distribution. The results of this study do not support the view that navigation, robotics, or patient-specific guides are mandatory in larger limbs or obese patients. The SPFI is a simple, reproducible metric that surgeons may find useful to stratify patients preoperatively, which may be more relevant to technical difficulty than BMI. In future, larger prospective studies are required to assess the effect of fat distribution on functional and patient-recorded outcomes measures.
